# The Prevalence and Diagnostic Patterns of Oral and Maxillofacial Lesions: A Seven-Year, Retrospective, Single-Center Cone Beam Computed Tomography and Histopathology Study in Saudi Arabia

**DOI:** 10.3390/jcm13247774

**Published:** 2024-12-19

**Authors:** Shadi Alzahrani, Tagreed Wazzan, Abdulaziz Almaghrabi, Abdulaziz Alkhudran, Hamzah Aljereb, Shadia Elsayed, Albraa B. Alolayan

**Affiliations:** 1 Department of Oral and Maxillofacial Surgery, Faculty of Dentistry, King Abdulaziz University, Jeddah P.O. Box 344, Saudi Arabia; saalzahrani12@kau.edu.sa; 2 Department of Oral and Maxillofacial Radiology, Faculty of Dentistry, King Abdulaziz University, Jeddah 21589, Saudi Arabia; twazzan@kau.edu.sa (T.W.);; 3 Faculty of Dentistry, King Abdulaziz University, Jeddah 21589, Saudi Arabia; abdulazizmg.1998@gmail.com (A.A.); mew.tesla.2021@gmail.com (A.A.); 4 Department of Oral and Maxillofacial Diagnostic Sciences, College of Dentistry, Taibah University, Medina 41477, Saudi Arabia; ssayed@taibahu.edu.sa

**Keywords:** oral pathology, maxillofacial lesions, prevalence, cysts, CBCT

## Abstract

**Objective:** To determine the prevalence of oral and maxillofacial lesions among patients at King Abdulaziz University from January 2016 to December 2022. **Methods**: This cross-sectional observational study included patients diagnosed with oral and maxillofacial intra-bony lesions based on radiological findings and confirmed by histopathological examination. The lesions were classified according to the fourth edition of the World Health Organization Classification of Head and Neck Tumors. **Results:** This study included 237 patients with a mean age of 31.53 ± 14.97 years, of which 45.1% were female. Most patients (46.7%) had mandibular lesions, followed by maxillary lesions (35.9%). Only 2.95% of the tumors were malignant. Odontogenic cysts were the most prevalent (65.40%), with radicular cysts and keratocytes being the most common types. The most prevalent tumor types were odontoma and ameloblastoma. The most malignant lesion was multiple myeloma. **Conclusions**: Our findings reveal that mandibular cystic lesions predominated, and emphasize the low incidence of malignancy in the study population. They provide valuable insights into the oral and maxillofacial lesion landscape at a high-volume tertiary care center.

## 1. Introduction

The prevalence of oral and maxillofacial lesions differs among populations. Understanding their prevalence patterns and characteristics might help physicians to make differential diagnoses and plan preventive measures. Oral and maxillofacial lesions comprise various disorders affecting oral and general health. They can cause various symptoms, including pain, infections, and mucosal abnormalities, or they may be completely asymptomatic [1]. Some oral lesions pose diagnostic challenges for oral health professionals, including general dentists and specialists [2,3]. The incidence of oral and maxillofacial lesions tends to increase with age, primarily due to physiological changes in the oral cavity. Their incidence also varies according to factors such as gender and anatomical location of the lesions [4,5,6]. Diverse diseases, from inflammatory to malignant, can exist due to the various histological tissue types in the region.

Various factors are used to classify these disorders [4,7]. Understanding the clinicohistopathological characteristics of these lesions is vital for correct diagnosis. The World Health Organization (WHO) introduced the latest Classification of Head and Neck Tumors in 2017, which plays a significant role in categorizing these lesions [4,6,7]. An essential subset of oral and maxillofacial lesions includes jaw cysts, which are incidentally discovered during routine radiographic examinations at the general dental office [8].

Cone beam computed tomography (CBCT) and histopathological examination have become essential in oral and maxillofacial surgery, due to their diagnostic accuracy and ability to provide high-resolution, detailed images of craniofacial structures. CBCT is particularly effective for evaluating bone quality, identifying lesions, assessing impacted teeth, and planning complex surgeries. Combining histopathological analysis with CBCT allows for a comprehensive pathology assessment. This dual approach enhances diagnostic precision and provides insights into the underlying etiology, enabling more effective treatment planning and outcome evaluation, and guiding clinicians in planning the extent of treatment and follow-up [9,10].

The prevalences of oral and maxillofacial lesions vary significantly among countries and regions, with reported rates ranging from 4.9% to 64.7% in the general population [11]. A Chinese study reported an overall prevalence of oral mucosal lesions of 10.8% [12]. Kaur et al. highlighted an increased prevalence of odontogenic tumors between 2005 and 2016 compared to previous years, which can be partly attributed to changes in classification criteria [13]. Sudarsan infrequently encountered odontogenic tumors at the studied hospital in Chennai, India, underscoring their relative rarity within this specific population [14]. Moreover, their prevalence can differ based on geographical factors and demographic characteristics such as age [15]. Despite this variation, epidemiological studies addressing the prevalence of oral and maxillofacial lesions in Saudi Arabia are needed. Therefore, this study aims to fill this gap by examining the prevalence of these lesions at a high-volume tertiary care center.

## 2. Materials and Methods

### 2.1. Study Design and Setting

This retrospective cross-sectional study was conducted between January 2016 and December 2022 at the Faculty of Dentistry of King Abdulaziz University (KAUFD) in Jeddah, Saudi Arabia. It focused on identifying and analyzing cases of oral and maxillofacial intra-bony lesions. Its chosen retrospective design enabled an extensive review of cases over this period, providing a robust sample size to analyze the trends, frequencies, and distribution patterns of various intra-bony lesions in the oral and maxillofacial regions.

### 2.2. Ethical Approval and Patient Confidentiality

This study was approved by the Research Ethics Committee at King Abdulaziz University (letter number: 009-01-23) and adhered to the principles in the Declaration of Helsinki. In order to maintain confidentiality, all the patients’ identities were kept anonymous during data extraction and processing, prioritizing patient safety and privacy and minimizing harm. Identifiers were removed, and unique alphanumeric codes were assigned to each case. The data were securely stored and made accessible only to authorized personnel directly involved in this study, further ensuring the confidentiality and ethical management of the patients’ information.

### 2.3. Inclusion and Exclusion Criteria

The following inclusion criteria were applied to create a comprehensive seven-year dataset for analysis: (1) radiographic evidence of intra-bony lesions in CBCT imaging of the oral and maxillofacial regions; (2) histopathological confirmation of histological intra-bony lesion diagnosis; (3) a complete set of records available in the institutional database, including demographic, clinical, radiological, and histopathological data; and (4) being diagnosed between January 2016 and December 2022.

The following exclusion criteria were applied to maintain data integrity and a focus on relevant intra-bony lesions in the maxillofacial region, ensuring a thorough analysis: (1) missing critical clinical, CBCT, or histopathological information; (2) inaccessible CBCT or histopathological records; and (3) lesions extending beyond the oral and maxillofacial regions, or findings unrelated to intra-bony pathology. The inclusion and exclusion criteria and the reasons for exclusion are presented in Table 1.

### 2.4. Diagnostic Imaging and Pathological Confirmation

The lesions were initially examined through CBCT, which was performed using standardized protocols by the university’s imaging department. The CBCT images were specifically reviewed to detect the presence, location, and initial characteristics of intra-bony lesions within the maxillofacial region. Two board-certified oral and maxillofacial radiologists interpreted the CBCT images to identify intra-bony abnormalities. After radiographic identification, a certified oral and maxillofacial pathologist confirmed the diagnosis through histopathological examination of biopsy samples collected from each case.

### 2.5. Reliability Assessment

Inter-observer reliability was evaluated by different examiners, who independently examined a random sample of 10% of the CBCT images and histopathological slides. In order to ensure diagnostic consistency, these assessments were performed over two weeks, and the intra-class correlation coefficient (ICC) was calculated. Intra-observer reliability of the same examiners reviewing the same sample was evaluated after two weeks. Both evaluations demonstrated significant agreement, with ICCs suggesting excellent reliability (ICC value > 0.75). Therefore, the study data exhibited significant consistency between examiners (inter-observer) and within the same examiner (intra-observer) over time.

### 2.6. Data Extraction

The data were collected in two stages. In the first stage, participants’ characteristics were retrieved from the institutional database, including their age, gender, pathology location (mandible, maxilla, or both), and biopsy results. In the second stage, the cases were pathologically classified based on the fourth edition of the WHO Classification of Head and Neck Tumors (Table 2) [16] as benign odontogenic tumors, malignant odontogenic tumors, odontogenetic cysts, and other bone-related and developmental lesions.

### 2.7. Statistical Analysis

Statistical analyses were performed using SPSS for Windows (version 21.0; IBM Corp., Armonk, NY, USA). Numerical variables were summarized as the mean ± standard deviation. Categorical variables were summarized as the number (percentage). A *p*-value of <0.05 was considered statistically significant.

## 3. Results

**Figure 1:**
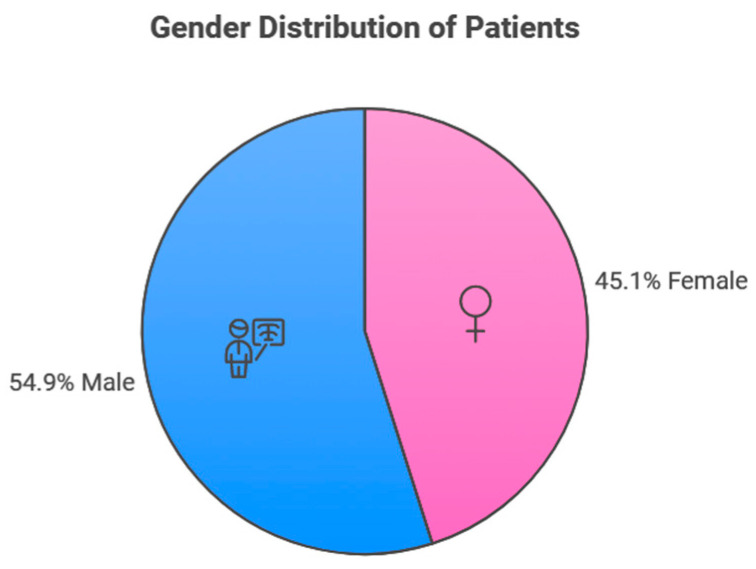
Gender distribution of the study populations.

**Figure 2:**
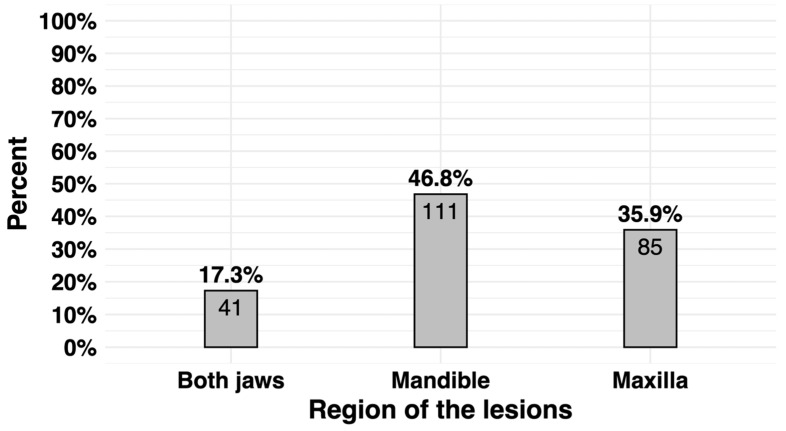
Participants’ lesion site distributions.

This study included 237 patients diagnosed with oral and maxillofacial lesions between January 2016 and December 2022, of which 107 (45.1%) were female and 130 (54.9%) were male (Figure 1). Their mean age was 31.53 ± 14.97 years (Figure 2).

### 3.1. Participants’ Lesion Sites

Among the participants, 111 (46.8%) had lesions in the mandible, 85 (35.9%) had lesions in the maxilla, and 41 (17.3%) had lesions in both the maxilla and mandible (Figure 1). The lesions were classified based on the 2017 WHO classification (Table 2), with the identified lesions presented in Table 3 and Table 4.

### 3.2. Benign and Malignant Odontogenic Tumors

Among the participants, 24 (10.12%) had benign odontogenic tumors. The most common benign lesion was odontoma (*n* = 12), followed by ameloblastoma (*n* = 8), ossifying fibroma (*n* = 2), cemento-ossifying fibroma (*n* = 1), and odontogenic myxoma (*n* = 1). In contrast, seven (2.95%) had malignant odontogenic tumors. The most common malignant lesion was multiple myeloma (*n* = 3), followed by squamous cell carcinoma (*n* = 2), chondroblastic osteosarcoma (*n* = 1), and spindle cell neoplasm (*n* = 1).

### 3.3. Odontogenic Cysts

Most of the patients (*n* = 155, 65.40%) had odontogenic cysts. The most common type was radicular cyst (*n* = 51), followed by keratocyst (*n* = 36), odontogenic cyst (*n* = 32), dentigerous cyst (*n* = 17), glandular odontogenic cyst (*n* = 6), simple bone cyst (*n* = 5), periapical cyst (*n* = 4), nasopalatine duct cyst (*n* = 3), and lateral periodontal cyst (*n* = 1).

### 3.4. Other Lesions

The remaining patients had other lesions (*n* = 51, 21.51%). The most common were periapical granuloma (*n* = 19), cemento-osseous dysplasia (*n* = 12), fibrous dysplasia (*n* = 6), osteomyelitis (*n* = 4), medication-related osteonecrosis of the jaw (*n* = 2), sialolith (*n* = 2), central giant cell lesion (*n* = 1), and sialoadenitis (*n* = 1).

## 4. Discussion

Our study addressed the prevalence and nature of oral and maxillofacial lesions at a high-volume tertiary care center at King Abdelaziz University in Saudi Arabia. Based on the latest WHO Classification of Head and Neck Tumors from 2017, its key findings indicated a high prevalence of odontogenetic mandibular cystic lesions, and emphasized the low incidence of malignancy in the study population. Its observations reveal the local epidemiology of these conditions, and provide valuable insights for healthcare providers [15,16].

Our study classified the oral and maxillofacial lesions based on the fourth edition of the WHO Classification of Head and Neck Tumors, released in 2017. This classification was chosen because it provides a comprehensive and up-to-date framework for categorizing lesions. It is widely recognized and accepted as a standard reference in the field, facilitating consistency and comparability in research across different regions and institutions. Different classifications can be used for oral bony lesions based on their clinical symptoms and etiology. The current fourth edition features some significant variations from the third edition, including a new classification for odontogenic cysts, a reclassification of odontogenic tumors, and some new entities [17]. Classifying and analyzing maxillofacial lesions are critical for guiding clinical practice and healthcare policy, facilitating an organized approach to therapy, and identifying significant geographical variations in incidence [18,19].

All the participants in our study underwent CBCT and had lesions confirmed by histopathological examination. Our data indicate a higher prevalence of odontogenic lesions in males than in females, which is consistent with findings from studies in Mexico, Ethiopia, and Turkey [20,21]. Interestingly, Alhindi et al. reported a higher percentage of affected females in a study at the same institution [22]. Notably, AlSheddi et al. found that these lesions predominantly affected males, with a male-to-female ratio of 1.4:1. Such gender variations merit further investigation, and might indicate different risk factors or tendencies for some genders to be more susceptible to particular lesion types [23].

Odontogenic cysts emerged as the predominant oral lesion in our study. Of these, radicular cysts were the most common, which is consistent with the findings of international studies [24,25]. Notably, the prevalence of odontogenic keratocysts was remarkably high in our study population, being the second most common type, which is consistent with the findings of Corso et al. in Italy [26]. Early diagnosis and management remain crucial, given the potential aggressiveness of keratocysts and their debated management strategies. Managing keratocysts requires more than enucleation and curettage, compared to a radicular or dentigerous cyst. Multiple adjuvant treatments have been used to decrease the recurrence rate with less morbidity. From enucleation and curettage alone, to peripheral osteotomy, the application of Carnoy’s solution and 5-fluorouracil in resection has been thoroughly investigated [27]. Odontogenic dentigerous cysts have been reported to be the second most common type of cyst, followed by radicular cysts, in various regions worldwide [20,21,28]. Another national study found that among odontogenic cysts, apical radicular cysts were the most common (64.3%). Despite their benign nature, odontogenic cysts can cause complications if not diagnosed and treated promptly, with some types showing locally aggressive behavior and a tendency to recur [29]. Depending on the lesion’s size and type, different maxillofacial lesions require different management approaches. While marsupialization followed by enucleation may be beneficial for larger multilocular lesions, enucleation is frequently effective for smaller unilocular lesions. Long-term follow-up is essential in many cases [30]. Excision and conservative therapy are among the other treatment choices, with bone grafting used when needed. The high occurrence of odontogenic cysts emphasizes the importance of efficient co-ordination between general dentists and oral and maxillofacial specialists, in order to properly recognize and manage these disorders [31,32].

Among benign odontogenic tumors, odontoma and ameloblastoma were found to be the most prevalent, a trend also noted by Izgi et al. [7]. In contrast, Almazyad et al. and AlSheddi et al. found ameloblastoma to be the most prevalent benign tumor, comprising 25–63% of cases [23,33]. Interestingly, a Malaysian study by Ismail et al. reported ameloblastoma as the most common type [27]. The incidence of malignant tumors was low in our study population. Only seven cases (2.96%) of malignant tumors were identified, aligning with the broader literature in Saudi Arabia, which has reported a relatively low incidence of oral cancer and potentially malignant lesions. A 10-year retrospective study in Southern Saudi Arabia found an oral malignancy incidence of 3.29% [33]. Other studies in the Jazan region reported that oral malignancies accounted for 15.8% of all malignancies [34,35]. Oral cancer screening during routine dental visits is critical for early identification and better patient outcomes. While 85% of dental practitioners undertake screenings, 48% conduct them annually, and 33% conduct them at every visit [36].

The complex anatomy of the posterior mandible can pose diagnostic challenges, potentially resulting in misdiagnosis and inappropriate treatment [37,38]. Timely identification and intervention can prevent potential complications and offer a better prognosis. A comprehensive oral examination allows dentists to detect, diagnose, and treat pathological conditions in the oral cavity. A thorough head and neck examination requires a solid foundation in normal anatomy and the ability to identify abnormal symptoms to avoid misdiagnosis, especially with untrained eyes [39,40]. Oral and maxillofacial lesions can present with clinical and histological features, from benign to malignant, requiring different treatment approaches. These lesions are significant, since they directly impact general and oral health. Their early diagnosis can prevent a delayed or inappropriate intervention. Our results emphasize that accurate diagnosis and treatment of oral bony lesions requires evaluation by a well-trained dental practitioner, which typically includes clinical and radiographic assessments and histological analysis.

Our study has some limitations. Firstly, it was conducted at a single center, limiting the generalizability of its findings. Secondly, it did not examine the distribution of maxillofacial lesion classifications over the study period. Therefore, future studies must investigate populations with diverse demographic and geographical characteristics. A multicenter approach would offer more diverse insights and a more comprehensive perspective on prevalence trends. The current observational study provides valuable trends regarding the prevalence and patterns of oral and maxillofacial conditions. However, it includes limited clinical data on lesion characteristics such as size, accompanying symptoms, and particular clinical presentations at the time of diagnosis. These elements could lead to a better knowledge of the lesions, and improve diagnostic and treatment planning processes. Future research should incorporate these clinical variables in order to expand on the findings of this study and permit more meaningful clinical linkages.

## 5. Conclusions

Our study revealed that odontogenic cysts, especially radicular cysts, were the predominant oral and maxillofacial lesions at KAUFD. The high percentage of keratocysts requires special attention and management. The prevalence of benign and malignant odontogenic tumors was 13.07%. Our findings emphasize the benefit of comprehensive clinical and radiographic assessments in oral health management. Further multicenter studies would provide a broader perspective on the regional prevalence of these conditions.

## Figures and Tables

**Table 1:** The inclusion and exclusion criteria and the reasons for exclusion.

Inclusion Criteria	Exclusion Criteria
Clear CBCT and a histopathologically confirmed diagnosis	Diagnosed outside KAUFD (*n* = 38, 17.75%)
Male or female	CBCT was conducted for another purpose (*n* = 132, 61.68%)
Age > 18 years	No CBCT reading and radiographic report (*n* = 16, 7.47) No histopathology results (*n* = 8, 3.73%) Treatment completed outside KAUFD (*n* = 20, 9.34%)

CBCT = cone beam computed tomography, KAUFD = Faculty of Dentistry of King Abdulaziz University.

**Table 2:** Pathological classifications in the World Health Organization (WHO) Classification of Head and Neck Tumors.

WHO Classification of Head and Neck Tumors: Odontogenic and Maxillofacial Bone Tumors.		
Benign Odontogenic Tumors		
Epithelial odontogenic tumors Ameloblastoma 1.1 Ameloblastoma, unicystic type 1.2 Ameloblastoma, extraosseous/peripheral type 1.3 Metastasizing ameloblastoma Squamous odontogenic tumor Calcifying epithelial odontogenic tumor Adenomatoid odontogenic tumor	Mixed epithelial and mesenchymal odontogenic tumors Ameloblastic fibroma Primordial odontogenic tumor Odontoma 3.1 Odontoma, compound type 3.2 Odontoma, complex type Dentinogenic ghost cell tumor	Mesenchymal odontogenic tumors Odontogenic fibroma Odontogenic myxoma/myxofibroma Cementoblastoma Cemento-ossifying fibroma
Malignant odontogenic tumors		
Odontogenic carcinomas Ameloblastic carcinoma Sclerosing odontogenic carcinoma Primary intraosseous carcinoma NOS Clear cell odontogenic carcinoma Ghost cell odontogenic carcinoma	Odontogenic carcinosarcoma	Odontogenic sarcoma
	Odontogenic cysts	
Odontogenic cysts of inflammatory origin Radicular cyst Inflammatory collateral paradental cyst	Odontogenic and non-odontogenic developmental cysts Dentigerous cyst Odontogenic keratocyte Lateral periodontal cyst and botryoid odontogenic cysts Gingival cyst Glandular odontogenic cyst Calcifying odontogenic cyst Orthokeratinized odontogenic cyst Nasopalatine duct cyst	

NOS = not otherwise specified.

**Table 3:** Lesion classifications.

Lesion Type	Number	Percentage
Benign odontogenic tumor	24	10.12%
Malignant odontogenic tumor	7	2.95%
Odontogenic cyst	155	65.40%
Other	51	21.51%
Total	237	100.00%

**Table 4:** The specific lesions according to the 2017 WHO classification.

Lesion Classification	Type	*n* (%)
Benign odontogenic tumors	Odontoma	12 (50%)
	Ameloblastoma	8 (33.3%)
	Ossifying fibroma	2 (8.3%)
	Cemento-ossifying fibroma	1 (4.2%)
	Odontogenic myxoma	1 (4.2%)
Malignant odontogenic tumors	Multiple myeloma	3 (42.9%)
	Squamous cell carcinoma	2 (28.8%)
	Chondroblastic osteosarcoma	1 (14.3%)
	Spindle cell neoplasm	1 (14.3%)
Odontogenic cysts	Radicular cyst	51 (32.9%)
	Keratocycst	36 (23.2%)
	Odontogenic cyst	32 (20.6%)
	Dentigerous cyst	17 (11%)
	Glandular odontogenic cyst	6 (3.9%)
	Simple bone cyst	5 (3.2%)
	Periapical cyst	4 (2.5%)
	Nasopalatine dust cyst	3 (1.9%)
	Lateral periodontal cyst	1 (0.6%)
Other lesions	Periapical granuloma	19 (37.2%)
	Cemento-osseous dysplasia	12 (23.5)
	Fibrous dysplasia	6 (11.8%)
	Osteomyelitis	4 (7.8)
	Medication-related osteonecrosis of the jaw	2 (3.9%)
	Sialolith	2 (3.95)
	Central giant cell lesion	1 (2%)
	Sialoadenitis	1 (2%)

## Data Availability

The original contributions presented in this study are included in the article. Further inquiries can be directed to the corresponding author.
